# Supplementary material for: Identification of Freezing-Responsive microRNAs and Their Targets in Chinese Jujube by Small RNA and Degradome Sequencing
Source: Int J Mol Sci. 2026 Sep 4;27(17):7912. doi: 10.3390/ijms27177912 (PMC13566890; doi:10.3390/ijms27177912)
Supplement: Supplementary file 1 [file ijms-27-07912-s001.zip › Supplementary Material S2/novel_miR_84.pdf]

Provisional ID	:	novel_miR_84
Score total	:	1.6
Score for star read(s)	:	-1.3
Score for read counts	:	0
Score for mfe	:	1.3
Score for randfold	:	1.6
Score for cons. seed	:	
Total read count	:	55
Mature read count	:	54
Loop read count	:	1
Star read count	:	0

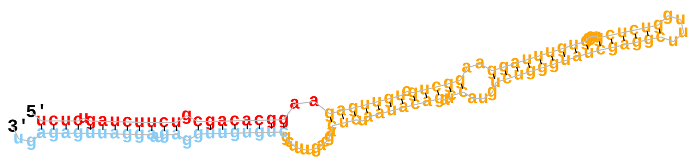

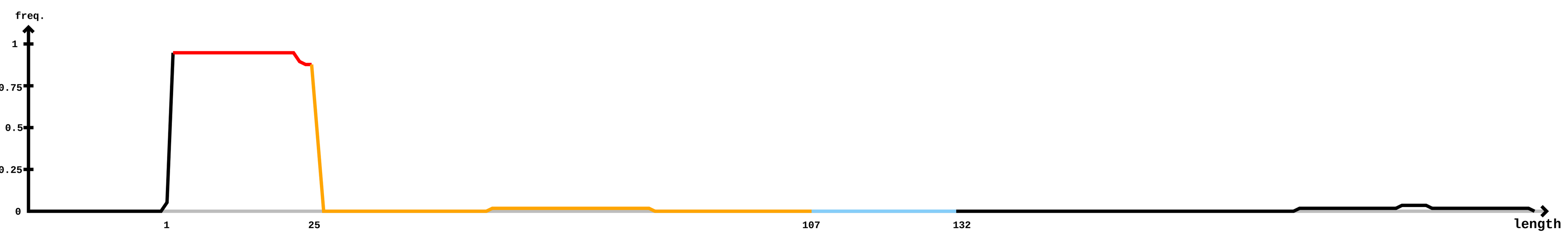

Mature Star

```
5'- uagauuucucagguaaggagauucucugaucuucugcgacacggaagaguugucgucggaaggauuuguggguugcucugguuucggagcuaugggucuguaccuagacauaaucucagcaguuuucgcuguguuggaguaaggauugagagucuugaucaugguuggaauuuggugcuaauuugcuaacuuauuuucuucacuccgguuaggaagauaggagagcaauggaggcaagaacuauuuugcg -3'   exp
    ((((((((((((((...(((((((((.((((((((.((((((((..(((((((.(((((..(((((((((....((((((....)))))))))))))))...))..))))))).)))...........)))))))).))..))))))))))))))).))).)))..))))))))...((...(((((..(((((((((((.((....)).)))))))))))..)))))...))((((((....)))))).   reads   mm   sample
    .....................uucucugaucuucugcgacacC...............................................................................................................................................................................   3   1   LCK-3
    ......................ucucugaucuucugcgacacgg..............................................................................................................................................................................   1   0   LT-2
    ......................ucucugaucuucugcgacacggaa............................................................................................................................................................................   27   0   LCK-2
    ......................ucucugaucuucugcgacacggaa............................................................................................................................................................................   23   0   LCK-1
    ...........................................................................cucugguuucggagcuaugggucugua....................................................................................................................................................   1   0   LT-3
    .................................................................................................................................................................................................................uaggaagauaggagagcaaugg...................   1   0   LT-3
    ..................................................................................................................................................................................................................................aauggaggcaagaacuauuuug..   1   0   LT-1
```
